# Review of the Effect of Natural Compounds and Extracts on Neurodegeneration in Animal Models of Diabetes Mellitus

**DOI:** 10.3390/ijms20102533

**Published:** 2019-05-23

**Authors:** Carmen Infante-Garcia, Monica Garcia-Alloza

**Affiliations:** 1 Division of Physiology, School of Medicine, Universidad de Cádiz, Edificio Andres Segovia. C/Dr. Marañon 3, 3er piso, 11002 Cádiz, Spain; carmeninfante@uca.es; 2 Division of Physiology, School of Medicine, Instituto de Investigación e Innovación en Ciencias Biomedicas de la Provincia de Cadiz (INiBICA), Universidad de Cádiz, 11002 Cádiz, Spain

**Keywords:** type 2 diabetes, inflammation, vascular damage, learning, memory, neuroprotection, natural extract, natural compound

## Abstract

Diabetes mellitus is a chronic metabolic disease with a high prevalence in the Western population. It is characterized by pancreas failure to produce insulin, which involves high blood glucose levels. The two main forms of diabetes are type 1 and type 2 diabetes, which correspond with >85% of the cases. Diabetes shows several associated alterations including vascular dysfunction, neuropathies as well as central complications. Brain alterations in diabetes are widely studied; however, the mechanisms implicated have not been completely elucidated. Diabetic brain shows a wide profile of micro and macrostructural changes, such as neurovascular deterioration or neuroinflammation leading to neurodegeneration and progressive cognition dysfunction. Natural compounds (single isolated compounds and/or natural extracts) have been widely assessed in metabolic disorders and many of them have also shown antioxidant, antiinflamatory and neuroprotective properties at central level. This work reviews natural compounds with brain neuroprotective activities, taking into account several therapeutic targets: Inflammation and oxidative stress, vascular damage, neuronal loss or cognitive impairment. Altogether, a wide range of natural extracts and compounds contribute to limit neurodegeneration and cognitive dysfunction under diabetic state. Therefore, they could broaden therapeutic alternatives to reduce or slow down complications associated with diabetes at central level.

## 1. Type 2 Diabetes Mellitus: Central Complications

Metabolic disorders include a broad range of alterations. Moreover, the terminology used to refer to many of the diseases and complications is confusing in many cases [1,2]. Among these, diabetes mellitus (DM) plays a preponderant role, due to its prevalence and societal and economical burden. In 2013 over 380 million people suffered diabetes and it is estimated that by 2035 there will be 592 million diabetic patients [3]. World Health Organization (WHO) defines DM as a chronic metabolic disease caused by inherited and/or acquired deficiency in the production of insulin by the pancreas, or by the ineffectiveness of the insulin produced. Such a deficiency results in increased concentrations of glucose in the blood, which in turn damage many of the body’s systems, in particular the blood vessels and nerves [4]. The two main forms of diabetes are type 1 diabetes (T1D) and type 2 diabetes (T2D), which account for >85% of the cases [3]. T1D and T2D differentially impact populations based on age, race, ethnicity, geography and socioeconomic status [5]. T1D is the most frequent type of diabetes in children and adolescents [6]. T1D patients suffer the destruction of over 90% of β-pancreatic islets, with consequent reduction of insulin and glycaemia control. On the other hand, T2D affects adults preferentially. However, the prevalence of T2D in adolescents and young adults is dramatically increasing [7]. T2D is characterized by an initial stage of insulin resistance. To compensate hyperglycaemia, β-pancreatic cells respond by increasing insulin production and establishing a prediabetic state. When exhausted β-pancreatic cells can no longer overproduce insulin, diabetes evolves. T2D is associated to a large list of risk factors, including familiar risk, previous gestational diabetes or life styles, among others [8]. 

While peripheral micro and macrovascular complications associated with T2D, such as neuropathies, retinopathies or nephropathies, have been widely studied [9], only in recent years attention has been paid to central complications associated with long-term metabolic alterations [10]. The mechanisms implicated have not been completely elucidated; however, cognitive impairment, vascular dementia, Alzheimer’s disease, stroke or anxiety/depression have been related to diabetes [1,11]. In this sense, the diabetic brain (with controlled or uncontrolled hyperglycemia) show brain injury with a wide profile of micro and macrostructural changes, leading to neurodegeneration, neurovascular deterioration, neuroinflammation and progressive cognition dysfunction [12,13,14,15,16,17,18,19]. However, the study of central complications associated with T2D has been probably hampered by the difficulty of the measurements [2], the lack of ideal animal models, or the fact that T2D is a complex disorder and, therefore, it is likely that multiple different, synergistic processes may interact to promote central alterations. Accordingly, the vast majority of the research are epidemiological studies in which T2D is identified as a risk factor for Alzheimer’s disease or vascular dementia [20,21,22,23]. Only a few studies have captured quality data regarding metabolic and cognitive status to allow reliable diagnosis of both T2D and dementia subtype. Main limitations are due to the fact that many of the studies rely on self reported diabetes, underestimating the prevalence by up to 50%, medical records are incomplete or may even include undiagnosed diabetics as control samples [2]. Moreover, patients with diabetes are often presumed to have dementia of vascular origin. However, the main limitation might be to determine the effects of medication, since treatments for T2D may also affect brain-associated complications [2]. Hence, in order to accurately delineate the pathogenesis of cognitive impairment in people with T2D, large-scale, prospective epidemiological studies are still required [24]. 

## 2. Natural Compounds and Central Complications in DM

The wide and countless number of natural compounds from plants, animals, fungi, microorganisms and other natural resources provides a rich and a unique source in the search of new drugs [25]. The potential health risk in the indiscriminate use of natural products cannot be obviated [26]. However, plant compounds, including different natural products (single isolated compounds) and/or natural extracts (including different compounds and/or secondary metabolites), have been long analyzed and assessed in relation with different pathologies. Usually, biological activity in plants’ natural extracts is mainly due to secondary metabolites. Plant secondary metabolites include two extensive categories: Nitrogen-containing compounds and those without it [27,28]. In line with these observations, several studies have shown a wide range of biological activities in these extracts, including anti-inflammatory [29,30], anti-microbial [31], anti-diabetic [18,32] or neuroprotective [27,33,34] properties, among others.

One of the most extensive group of secondary metabolites in the plant kingdom are polyphenols [35]. Structurally, they are characterized by the presence of at least one hydroxyl functional group (-HO) linked to an aromatic ring [36]. Polyphenols classification is referred to the number of phenol rings in the molecule, and the main subgroups include phenolic acids, stilbenes, flavonoids, coumarins and lignans [35]. The wider group of polyphenols in plants is represented by flavonoids, which account for over 10,000 different compounds [28,35]. As other natural compounds, flavonoids have shown several properties including antioxidant, neuroprotective [37] or anti-diabetic [38,39,40] effect. Another particularity of polyphenols is their role in human nutrition, which extends their utility, including not only a pharmacological, but also a nutritional perspective. This singularity of polyphenols contributes to further study of these compounds in other fields, such as human diet supplements [35,41]. 

As mentioned above, DM, or even prediabetes state, are associated with an increased risk to suffer neurodegenerative diseases, specially vascular dementia and Alzheimer’s disease [42,43]. Therefore diabetic control may be an important and modifiable risk factor to reduce diabetes-associated neurodegeneration [44]. In this sense, while the number of articles published worldwide in relation with antidiabetic natural products is growing each year, most of them focus on metabolic control and related alterations [45]. On the other hand, studies on the effect of natural products and extracts on central complications associated with DM are more scarce. This is mainly due to the difficulty to identify individual components in complex extracts, the capability of different molecules to cross the blood brain barrier, or even discriminate the direct effect of diabetes on the pharmacokinetics, bioavailability and brain distribution of the compounds and metabolites [46]. However, given the well established complications of DM on the central nervous system, there are different targets of interest that may be covered by natural compounds, including vascular damage, neuroinflammation, neurodegeneration or cognition. Following this idea, several natural compounds and extracts have been reported to show neuroprotective effects [34,38].

### 2.1. Natural Compounds and DM-Related Vascular Injury

#### 2.1.1. Vascular Damage and DM

Vascular complications are the leading cause of morbidity and mortality in diabetic patients. Vascular alterations are derived from the chronic hyperglycemic state that can affect both large and small blood vessels, characterizing diabetes macro and microangiopathy, respectively [47]. Several vascular alterations including irreversible non-enzymatic glycation of proteins, cellular redox potential alteration, increased oxidative stress or inflammatory response, as well as endothelial dysfunction or hypercoagulability contribute to vascular abnormalities associated to DM [47,48,49]. These underlying alterations may support the fact that diabetic patients present arterial stiffness as well as increased risk of atherosclerosis and cerebral stroke [50,51,52]. In line with these observations, previous studies have reported that DM patients have smaller brain volumes and white matter lesions, which have been associated to neurovascular unit dysfunction and blood brain barrier alterations. In this context T2D could cause loss of homeostasis of the cerebral microenvironment, leading to vascular damage and astrocyte alterations [53]. In addition, preclinical studies in diabetes animal models have shown exacerbated neurovascular damage, and ultrastructural abnormalities, characterized by mural endothelial cell tight and adherens junction or perycite attenuation or loss [54]. Likewise, studies in mouse models reveal brain overspread microbleeding, reproducing small vessel disease [55,56]. DM not only exacerbates neurovascular damage but also hinders the brain repair process, likely contributing to the impairment of stroke recovery [57]. In this sense, in vitro and in vivo experimental models have showed that the integrity of the blood brain barrier is affected in diabetic conditions [58,59,60]. Concretely, diabetes disrupts the blood brain barrier endothelium by downregulation of cell junction proteins [61,62,63] and upregulation of integrin expression [64,65], leading to abnormal vascular permeability [66,67]. In addition, this effect might be mediated by oxidative stress, which induces blood brain barrier disruption through osmotic damage and pericyte loss [68], ultimately leading to the leak of toxic substances and further damage to the nervous structures [69]. Interestingly, microvascular alterations seem to be present also in prediabetic animal models [70], suggesting that early hyperinsulinemia and insulin resistance are enough to induce vascular damage. 

#### 2.1.2. Natural Compounds and Extracts in Vascular Damage Associated with DM

**Figure 1:**
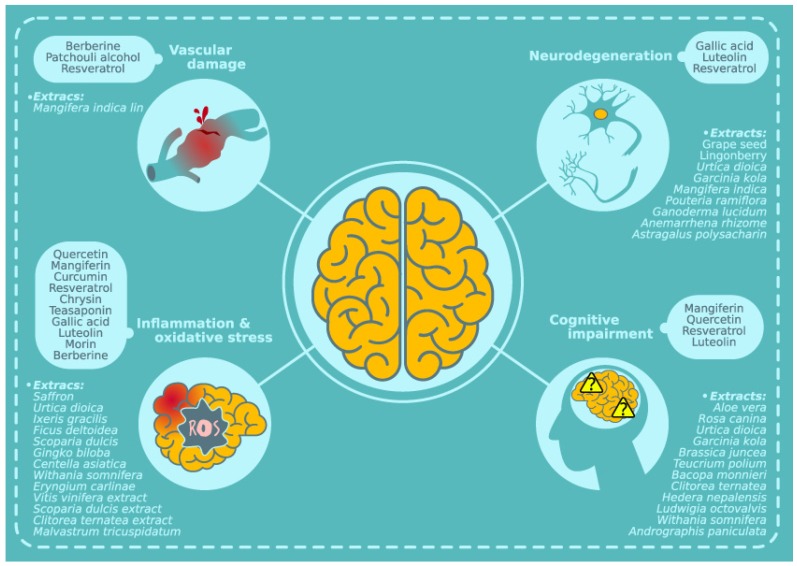
Central activities of natural compounds and extracts.

In order to try and reverse many of these complications different natural compounds and extracts have been used in animal models. In this sense berberine, a protoberberine present in a number of medicinal plants [71], and the main active component of *Coptis chinensis French* has been used for years, and studies in patients have shown its capability to regulate glucose and lipid metabolism [72]. Moreover, at central level it has also been reported that berberine may reduce diabetes induced ectopic expression of miR-133a in endothelial cells, which is involved in endothelial dysfunction in DM. In addition, berberine may inhibit acetylcholine-induced vasorelaxation in the middle cerebral artery, guaranteeing better blood supply to the brain in streptozotozin (STZ)-treated rats, as a T1D model [73]. It has also been reported that patchouli alcohol, a natural tricyclic sesquiterpene in the traditional Chinese herb Pogostemonisherba [74], reduces ishcemia/reperfusion damage after middle cerebral artery occlusion in ob/ob mice by limiting infarct volume, protecting blood brain barrier function and decreasing inflammatory markers [74]. In line with these observations, *Mangifera indica Lin* extract, rich in natural polyphenols, reduces spontaneous central bleeding detected in db/db mice. While the actual size of the microbleeds is not affected, *Mangifera indica* extract reduces the appearance of new vascular lesions [18]. In addition, poor cerebral perfusion may contribute to cognitive impairment in diabetic state and resveratrol, a natural phenol isolated from plants like *Polygonum cuspidatum*, *Paeonia lactiflora* and *Vitis amurensis*, among others [75], may improve neurovascular coupling capacity in T2D patients [76] and reduce blood brain barrier permeability and vascular endothelial growth factor expression in the hippocampus of diabetic rats [77] (Table 1 and Figure 1).

### 2.2. Natural Compounds and Neuroinflammation Associated with DM

#### 2.2.1. Brain Neuroinflammation and DM

Inflammation is an immune response against several conditions including disease and infection. Acute inflammatory events are resolved efficiently and inflammation levels return to baseline in physiological conditions. However, in chronic inflammation the resolution phase is not achieved due to excessive pro-inflammatory signalling and it can provoke relevant detrimental effects [78]. Following this idea, insulin resistance and diabetes are closely associated with chronic inflammation [79]. Moreover, the finding two decades ago that proinflammatory cytokines like tumor necrosis factor-α (TNF-α), among others, are overexpressed in adipose tissue of obese mice provided a relation between obesity, diabetes and chronic inflammation [79,80,81].

Inflammation in the central nervous system is complexly regulated and astrocytes [82], blood inflammatory cells and even neurons seem to participate and mediate inflammation in the injured brain. However, microglia still play the most significant role at this level [83]. Microglia are a specific type of macrophage in the brain; they are held without external replenishment and they are not in contact with plasmatic proteins, which contributes to keep an immupriviledged environment in the central nervous system [84]. The classical dual role of microglia as a protective (with a typical anti-inflammatory profile) or damaging agent (with a proinflammatory response) has been recently reviewed and microglia-mediated responses seem to be more prone towards neuronal survival, regeneration [85] and overall neuroprotection [86]. The role of microglia in neurodegenerative diseases has been long studied and they also seem to be highly activated in metabolic disease models, ranging from prediabetic [87], T1D [88], T2D [55,56] models, or even diabetic mothers offspring [89]. Under diabetic conditions, hyperglycemia leads to increased mitochondrial respiration in pericytes, astrocites as well as endothelial cells [90]. This causes an increase in the production of reactive oxygen species that may consequently lead to neurovascular damage and blood brain barrier dysfunction, contributing to the inflammatory process. Increased levels of reactive oxygen species may also affect protein fuction, signaling pathways or induce upregulation of inflammatory cytokines [90]. Therefore, previous studies have shown that, in metabolic alterations, microglia mediated neuroinflammation may contribute to the neurodegenerative process by promoting the release of cytokines and chemokines including TNF-α [91,92]. In line with these ideas, studies in patients with metabolic disorders have detected a decrease in mRNA levels of the IL10-mediated anti-inflammatory defense, while iNOS-mediated inflammatory activity seems to be favored in the cortex from obese patients [93]. 

#### 2.2.2. Effect of Natural Compounds on DM-Related Inflammation

Antioxidant and anti-inflammatory activities are probably the most widely explored roles of natural compounds and extracts [30,94,95]. Following this idea, many studies have previously used products of natural origin to counterbalance oxidative stress, neuroinflammation and alterations in related markers and cytokines. Even though the role of flavonoids in neuroprotection might be due to different mechanisms of action it is mediated, at least in part, by direct scavenging of free radicals as antioxidant action [35,96]. Several plants extracts constitute a relevant source of polyphenols. While in many cases they share common mechanisms and show potent anti-inflammatory and antioxidant activities, not all of them have been completely characterized. Concretely quercetin, present in many fruits and vegetables, may enhance glyoxalase pathway activity, inhibit advanced glycation end products (AGEs) formation and reduce oxidative stress [97]. Quercetin is a flavonoid present in a wide variety of plants, including *Rosa canina*, *Opuntia ficusindica* and *Allium cepa* [75]. Oral administration of quercetin to diabetic rats has shown antioxidant effects, increasing superoxide dismutase (SOD) and catalase activity, while also restoring the blood levels of vitamin C and E, which finally contribute to ameliorate the diabetes-induced in oxidative stress [98]. On the other hand, it has been described that quercetin also protects neuronal PC12 cells against high-glucose-induced oxidative stress, inflammation and apoptosis [99]. While the final underlying mechanisms involved in quercetin neuroprotective effects are not completely known, a recent study has shown that neuroprotection might mediated by phosphorylation regulation of Nrf2/ARE/glyoxalase-1 pathway in central neurons under chronic hyperglycemia, reducing AGEs and oxidative stress [38]. In line with these observations mangiferin, which is mainly present in *Mangifera indica L.* but also in Chinese herbal medicines like *Rhizoma Anemarrhenae* and *Rhizoma Belamcandae*, has anti-inflammatory [100] and antioxidant [100,101] activities. Mangiferin also enhances the function of glyoxalase-1 through activation Nrf2/ARE pathway in neurons exposed to chronic high glucose [101]. In addition, *Mangifera indica L.* extracts with a high content in mangiferin and quercetin reduce microglia activation and associated inflammation in db/db mice after long-term treatment [18].

On the other hand curcumin, a bright yellow compound isolated from the rhizome of *Curcuma longa* [75] has shown neuroprotective effects in diabetic rats reducing blood glucose, oxidative stress markers and astrocyte activation in the hippocampus [102]. A recent study has reported the potent neuroprotective effect of J147, a novel curcumin derivative developed to increase curcumin bioavailability and blood brain barrier permeability [103]. J147 reduces inflammation by decreasing TNF-α pathway activation and several other markers of neuroinflammation in mice treated with STZ [103], supporting that different curcumin extracts and derivates are potent antioxidants with the capability to limit associated central complications in diabetes. Resveratrol has a well established antioxidant activity. It reduces astrocytic activation as well as TNF-α, IL-6 transcripts the hippocampus of diabetic rats [77]. Resveratrol also normalizes malonedialdehyde and oxidezed glutathione levels in diabetic rats and it strengthens the action of antioxidants enzymes SOD and catalase [104]. *Ficus deltoidea* leaf extract also increases SOD and glutathione peroxidase values, while reducing thiobarbituric acid reactive substances [105]. Similar outcomes have been reported for saffron extracts with antidiabietic activity, which also modulate antiinflamatory pathways at central level [106]. Likewise, *Scoparia dulcis* plant extract also increases activities of plasma SOD, catalase or glutahione peroxidase or glutathione-S-transferase while reduces gluthatione in the brain from STZ diabetic male rats [107]. Similar outcomes have been described for chrysin, a flavonoid isolated from *Oroxylum indicum*, *Passiflora caerulea*, *Passiflora incarnata*, *Teloxys graveolens* and *Artocarpus heterophyllus* that also ameliorates oxidative stress by reducing catalase levels, SOD and glutathione in the cerebral cortex and hippocampus of diabetic rats [108]. 

One of the most widely studied preparations is Gingko biloba extract EGb 761, which has been described to scavenge reactive nitrogen and oxygen species, as well as peroxyl radicals [35,96,109]. A similar scavenging effect has been described for green tea extracts [35,110]. In this sense, tea extract, teasaponin, also reduces proinflammatory citokines and inflammatory signaling in the hypothalamus from mice on high fat diet [111]. For its part, *Clitorea ternatea* leaf extract, has showed protection against oxidative stress increasing SOD, total nitric oxide, catalase and glutathione levels in the brain of diabetic rats [112]. Similar antioxidant effects have been reported for grape seed extracts (*Vitis vinifera* sp.), rich in flavonoids like proanthocyanidins, showing beneficial effects on oxidative stress in the hippocampus of STZ-induced diabetes rats, to a larger extend than a classical antioxidant as viatamin E [113]. The expression of inflammatory TNF-α, and NF-κB genes are significantly reduced and other studies have also reported the role of grape seed extract in modulating AGEs/RAGE/NF-kappaB inflammatory pathway in the brain [114]. *Urtica dioica* leaves extract, rich in scopoletin, rutin, esculetin and quercetin, has also shown antioxidant and anti-inflamatory activities in the hippocampus from STZ-induced diabetic mice [115,116]. In addition, the number of astrocytes in the hippocampus from diabetic rats is reduced after treatment with *Urtica dioica* extract, supporting its anti-inflammatory role at different levels [117]. 

Gallic acid, is a type of phenolic acid, which is isolated from several plants including *Phaleria macrocarpa, Peltiphyllum peltatum*, and *Pistacia lentiscus*. Gallic acid may inhibit hippocampal neurodegeneration via its potent antioxidant and anti-inflammatory effects in diabetic rats [118]. Similarly, *Scoparia dulcis* extract also reduces thiobarbiyutic acid reactive substances and hydroperoxides formation in the brain from diabetic rats, supporting its role in protection against lipid peroxidation induced membrane damage [107]. Luteolin can also reduce neuroinflammation by reducing plasma and brain cytokines in a prediabetic mouse model [119]. Moreover, similar antioxidant and anti-inflammatory effects have been reported for luteolin in diabetic mice [120]. Other studies in prediabetic models have shown a protective role for *Withania somnifera*, which may reduce gliosis and microgliosis as well as expression of inflammation markers such as PPARγ, iNOS, MCP-1, TNF-α, IL-1β, and IL-6 [121]. In line with these observations, oral administration of an hexanic extract of *Eryngium carlinae* inflorescences to diabetic rats not only reduced glucose levels but also limited overall oxidation, by reducing lipid peroxidation, protein carbonylation and reactive oxigen species production, while increasing catalase activity in the brain [122]. Morin is another flavonoid isolated from *Maclura pomifera* and *Maclura tinctoria*, with similar properties [123,124]. Additionally, the flavonoid rutin has also shown antioxidant properties in the diabetic rat retina [125]. In line with these observations, berberine has been shown to reduce oxidative stress and astrogliosis in the hippocampus from diabetic rats [126]. A natural extract from *Centella asiatica,* rich in ascorbic acid, asiatic acid, oleanolic acid, stevioside, stigmasterol and α-humulene protects diabetes tissues from stress via antioxidant and anti-inflammatory mechanisms eliciting brain reduced levels of malondialdehyde, TNF-α, IFN-γ, IL-4 or IL10 [127]. Similar outcomes have been reported for *Ixeris gracilis* extract used in mice with alloxan-induced diabetes [128]. Specific assessment of mitochondrial status in STZ-induced diabetes has also revealed the capacity of *Malvastrum tricuspidatum* extract to restore oxidative damage [129] (Tables 1 and 2, and Figure 1).

### 2.3. Natural Compounds and Brain Neurodegeneration in DM

#### 2.3.1. Neurodegeneration in Diabetic Brain

A wide range of clinical [15,19,130,131] and preclinical studies [70,88,132] have shown an association of prediabetes and diabetes with brain atrophy. In this sense, magnetic resonance studies have shown that both T1D and T2D patients have reduced grey matter density and white matter lesions, as well as cortical and hippocampal atrophy [133,134]. However, it seems that brain atrophy is more severe in T2D patients, probably given that this population is older on average [135,136,137]. As previously pointed out, the prediabetes process seems to be enough to induce brain atrophy in patients [138] and synaptic loss is also detected in animal models when prediabetes is combined with other central complications [132]. Likewise, animal models of metabolic alterations show neuronal simplification, synaptic alterations [44], reduced neuronal density and overall brain atrophy [55,56].

Neurodegeneration in diabetes is mediated by multiple neuropathogenic factors including hyperglycemia mediated damage, but also hypoglycemic episodes, cerebrovascular alterations or insulin derregulation in the brain or among others [139]. In this sense, dysfunction of insulin/insulin receptor mediated signaling might be responsible for alterations in synaptic plasticity, cognition and memory [139,140]. Once more, oxidative stress mediated by free radicals is related with the diabetes neurodegenerative process [141], given that hyperglycemic state reduces antioxidants levels and consequently increases the production of free radicals [139]. Neurons are especially vulnerable to oxidative stress and this can induce mitochondrial oxidative damage, resulting in apoptosis and/or necrosis [142]. On the other hand, several proteins implicated in neurodegeneration, such as tau protein, which is hyperphosphorylated in diabetic mouse models, may also underlie neuronal death [70,88,143]. In overall terms, neurodegeneration is perceived as a cause of cognitive dysfunction observed in diabetes conditions.

#### 2.3.2. Effect of Natural Compounds and Extracts on Brain Neurodegeneration Associated with DM

The majority of the studies on natural compounds and extracts have focused on their antioxidant and anti-inflammatory activities. However, neurodegeneration is a multifactorial pathogenic process and it is feasible than various, concomitant underlying mechanisms are responsible for their final neuroprotective effect. In this sense, polyphenols are able to modulate the activity of multiple involved targets, which contribute their pleiotropic effects (anti-inflamatory, antioxidant or inmunomodulatory) [144], and, indeed, phenolic compounds have shown their neuroprotective role in vitro, in animal models and in clinical studies [145,146,147,148]. In line with these observations, flavonoids are not only implicated in scavenging of free radicals and reducing oxidative stress [35,96], but they can also modulate brain signaling cascades implicated in neuronal apoptosis, alter the expression of specific genes and modify mitochondrial activity [149]. 

*Mangifera indica* extract has shown its capability to limit brain atrophy in db/db mice. Cortex and hippocampus are largely preserved after long-term administration [18]. Interestingly, oral treatment with *Mangifera indica* also reduces tau hyperphosphorylation, an early marker of neuronal damage, and it also preserves compromised neuronal population in this model [18]. In line with these observations quercetin has also been shown to protect neuronal PC12 cells against high-glucose-induced oxidative stress, inflammation and apoptosis [99], as described for gallic acid in diabetic mice [118]. Curcumin protects against structural alterations of the hippocampus associated with diabetes, by reducing disorganization of small pyramidal cells in CA1, cellular loss in the pyramidal cells of CA3 and degenerated granule cells in the dentate gyrus [102]. In addition, curcumin derivate, J147, has been shown to upregulate nervous system development functions in diabetic mice. Moreover, functions related with neuron growth, such as proliferation, axon growth and long-term potentiation are the most significantly changed [103]. Luteolin also shows neuroprotective activity by increasing the levels of brain-derived neurotrophic factor, the action of synapsin I and postsynaptic density protein 95 in the cortex and hippocampus from mice on high fat diet [119]. Likewise, resveratrol has also been widely assessed and chronic treatment improves neuronal injury, not only through attenuation of oxidative stress and neuroinflammation, but also by reducing synaptic loss and increasing synaptic plasticity markers SYN and GAP-43 [150], as well as by inhibiting hippocampal apoptosis through the Bcl-2, Bax and caspase-3 signaling pathways in STZ-induced diabetic rats [151]. Gallic acid may inhibit hippocampal neurodegeneration in diabetic mice not only through its potent antioxidant and anti-inflammatory activities, but also due to its anti-apoptotic properties [118]. 

Other mechanisms of action have been presented for different compounds and extracts, many of which have focused on the hippocampus, a key area in learning and memory. In this sense *Astragalus Polysacharin* extract may upregulate phosphorylation levels of N-methyl-d-aspartate receptor, calcium/calmodulin-dependent protein kinase II and cAMP response element-binding protein, as well as reduce the number of dead cells in the CA1 region of the hippocampus from STZ-treated diabetic rats [152]. On the other hand, antioxidants present in bilberry fruits, rich in anthocyanins, influence the morphology of and possibly exhibit beneficial and neuroprotective effects on hippocampal neurons during diabetes [153]. *Pouteria ramiflora* extract administration to STZ-treated rats exerts hippocampal neuroprotection by restoring myosin-Va expression and the nuclear diameters of pyramidal neurons of the CA3 and the polymorphic cells of the hilus [154]. In a T1D rat model, *Garcinia kola* seeds limit neuronal loss in regions involved in cognitive and motor functions, including the motor cortex, the medial septal nucleus an cerebellar Purkinje /granular cell layers [155]. *Urtica dioica* leaves extract also seems to exert it neuroprotective activities by modulating different pathways. It downregulates iNOS, while it upregulates BDNF, TrKB, cyclin D1, Bcl2, autophagy5 and autophagy7 mRNA expression and reduces TNF-α expression in diffrent hippocampal regions. In addition, an overall reduction of neuronal damage and DNA fragmentacion has been observed in the hippocampus from diabetic mice [156]. Other studies have also shown that *Urtica dioica* extract may limit granule cell loss of the dentate gyrus from young diabetic rats. While the positive effect is not observed when the extract is used preventively, it seems to ameliorate hippocampus cell loss when used as a treatment [157]. Similar outcomes have been observed after ginger extract administration, in combination with insulin, to male diabetic rats, showing changes in the expression of cyclin D1 gene and reducing apoptosis in hippocamapal cells [158]. Apart from its well established antioxidant activity, grape seed extract reduces caspases 3 and 9 expression in the hippocampus, ameliorating apoptosis in diabetic rats [113]. Another way of maintaining hippocampus integrity has been observed with an aqueous extract of *Anemarrhena rhizome*, capable of increasing cell proliferation and neurpeptide Y expression in the dentate gyrus from diabetic rats [159]. Lingonberry extract also exerts neuroprotective activity in diabetic rats by reducing oxidative stress, but also by restoring the density of purinergic receptors in the cortex [160]. In addition, in T2D mice with cerebral ischemic injury, chronic treatment with a water-soluble extract from the culture medium of *Ganoderma lucidum* mycelia reduced neuronal cell death and vacuolation in the ischemic penumbra, with reduced number of TUNEL, cleaved caspase-3 cells and the expression of receptor-interaging protein kinase 3 mRNA and protein, confering resistance to apoptosis and necroptosis [161] (Tables 1 and 2, and Figure 1).

### 2.4. Natural Compounds and Cognitive Impairment in DM

#### 2.4.1. Cognitive Dysfunction Associated with Diabetes

Substantial epidemiological evidence supports that cognitive dysfunction is a common complication of diabetes [162,163,164]. It has been estimated that 20–70% of patients with DM show cognitive decline, and 60% present at higher risk of dementia [11,12]. Following this idea, it is noteworthy that even prediabetic adults shown accelerated cognitive decline, associated with smaller total brain tissue volume [131]. Different stages of cognitive dysfunction have been associated with diabetes, depending on affected cognitive features, age or prognosis, andprobably with different underlying mechanisms [165,166,167]. Previous studies in patients have reported a wide range of diabetes-associated cognitive decrements ranging from subtle changes in cognitive function (that might give rise to cognitive complaints, but should not affect activities of daily life) and mild cognitive impairment, to severe forms like dementia [162,168]. Several factors, including vascular injury, insulin resistance, inflammation and depression, are potential risk factors for cognitive dysfunction in diabetic patients [168,169,170]. These data are also supported by studies in animal models, where severe cognitive impairment is observed in diabetic animal models that are also dependent on the model under study, the age and evolution of the disease [70,171].

#### 2.4.2. Effect of Natural Compounds and Extracts on Cognitive Impairment Associated with DM

As previously discussed, the mechanisms of action of natural compounds and extracts remain largely elusive, and it is feasible that a combination of different positive effects, including antioxidant, anti-inflammatory, vascular protection, antiapoptotic or proregenerative activities are responsible for observed beneficial effects in DM associated cognitive alterations. Concretely, mangiferin has been shown to counterbalance learning and memory impairments in diabetic rats, treated with STZ, when assessed in the Morris water maze [172]. Similarly, db/db mice on long-term *Mangifera indica* extract, with a high content of mangiferin, significantly improve their performance in the Morris water maze [18]. Moreover, episodic memory alterations are also ameliorated in a very demanding version of the new object discrimination test, and “what”, “where” and “when” paradigms are significantly improved [18]. Quercetin also ameliorates STZ-induced spatial learning and memory impairment in the Morris water maze [173,174], reducing the time spent in target quadrant in the test trial and increasing escape latendcy in the elevated plus maze. Similar results have been reported when chrysin [108] or *Andrographis paniculata* extract [175] are administered to STZ-treated rats. Similar outcomes have been reported when *Hedera nepalensis* extract is administered to STZ-aluminium trichloride rat model [176]. Likewise, grape seed proanthocyanidin extract [177], kola nut extract [178] or *Garcinia kola* seeds [155] also improve cognitive impairment in diabetic rat models. *Andrographis paniculata* extract, enriched in andrographolide, improves cognitive function in STZ-treated rats and the effect seems to be mediated by reducing oxidative stress and acetylcholinesterase activity [175]. Similar underlying mechanisms have been described for *Clitorea ternatea* leaves extract, which also improve spatial working memory, spatial reference memory, and spatial working-reference in the Y maze, the Morris water maze and radial arm maze, respectively, in diabetic rats [112]. In addition, studies with *Brassica juncea* extract [179] or resveratrol [150] have reported positive effects on learning and memory in diabetic rats. Equally, hydroalcoholic extract of *Teucrium polium* also limits cognitive impairment in the passive avoidance test while reducing oxidative stress markers in diabetic rats [180]. In addition, cognitive impairment is ameliorated in mice models after administration of *Rosa canina* hydro-alcoholic extract [181] or *Ludwigia octovalvis* extract [182]. Other studies on diabetic mice have shown that *Flos puerariae* extract also improves cognitive impairment after STZ administration, by reducing oxidative stress and restoring cholinergic activity (enhancing cholinacetyltransferase and alleviating acetylcholinesterase activities) in the the cortex from STZ-treated mice [183], and similar outcomes have been reported with *Withania somnifera* and *Aloe vera* extracts [184]. *Bacopa monnieri* [185] and *Urtica dioica* [115,186] extracts restore memory deficits in different diabetic mouse models. Additionaly, cognitive impairment in early metabolic alterations, such as prediabetic mice on a high fat diet, improve in the Morris water maze and the step-through task after luteolin [119] or *Ludwigia octovalvis* extract administation [182] (Table 1 and Table 2, and Figure 1).

Conclusions: Altogether, natural components and extracts show antioxidant and anti-inflammatory activities at central level, as well as a relevant capacity to reduce vascular damage, contributing altogether to limit neurodegeneration and cognitive derived alterations. Therefore, while the ultimate underlying mechanisms remain largely unknown, they could contribute to expand therapeutic options to treat or reduce central complications associated with DM. 

## Figures and Tables

**Table 1:** Natural compounds and extracts with activity at central level associated with metabolic disorders.

Natural Compound	Action	Plant Source	References
Berberine	Regulation of glucose and lipid metabolism. Reduction of diabetes induced ectopic expression of miR-133a involved in endothelial dysfunction associated with DM. Inhibition of acetylcholine-induced vasorelaxation in the middle cerebral artery → better blood supply to the brain in STZ-treated rats. Reduction of oxidative stress and astrogliosis in the hippocampus from diabetic rats.	*Coptis chinensis* French and others.	[72,73,126]
Patchouli alcohol	Reduction of ischemia/reperfusion damage after middle cerebral artery occlusion in ob/ob mice by limiting infarct volume, protecting blood brain barrier function and decreasing inflammatory markers.	Pogostemonisherba	[74]
Resveratrol	Improvement of neurovascular coupling capacity in T2D patients. Reduction of blood brain barrier permeability and VEGF expression in the hippocampus of diabetic rats. Restriction of astrocytic activation as well as TNF-α, IL-6 transcripts the hippocampus of diabetic rats. Normalization of malonedialdehyde and oxidezed glutathione levels in diabetic rats and strengthening of the action of antioxidants enzymes SOD and catalase. Improvement of neuronal injury by attenuation of oxidative stress and neuroinflammation, and by reducing synaptic loss and increasing synaptic plasticity markers SYN and GAP-43, as well as by inhibiting hippocampal apoptosis through the Bcl-2, Bax and caspase-3 signaling pathways in STZ-induced diabetic rats. Protection against learning and memory alterations in diabetic rats.	*Polygonum cuspidatum*, *Paeonia lactiflora* and *Vitis amurensis*, among others	[75,76,77,104,150,151]
Quercetin	Enhancement glyoxalase pathway activity, inhibition of AGEs formation and reduction of oxidative stress. Increase of SOD and catalase activities, restoring blood levels of vitamin C and E and ameliorating diabetes-induced oxidative stress. Protection of neuronal PC12 cells against high-glucose-induced oxidative stress, inflammation and apoptosis. Improvement in learning and spatial memory in the Morris water maze.	*Rosa canina*, *Opuntia ficusindica* and *Allium cepa*	[38,75,97,98,99,173,174]
Mangiferin	Improvement of the function of glyoxalase-1 through activationNrf2/ARE pathway in neurons exposed to chronic high glucose. Protections against learning and memory impairments in diabetic rats, treated with STZ.	*Mangifera indica Lin*, *Rhizoma Anemarrhenae* and *Rhizoma Belamcandae* among others	[100,101,172]
Curcumin	Neuroprotective effects in diabetic rats reducing blood glucose, oxidative stress markers and astrocyte activation in hippocampus. Protection against structural alterations of the hippocampus associated with diabetes.	*Curcuma longa*	[75,102]
J147 curcumin derivative	Increase of curcumin bioavailability and blood brain barrier permeability. Reduction of inflammation by decreasing TNF-α pathway activation and several other markers of neuroinflammation in mice treated with STZ. Upregulation of nervous system development functions in diabetic mice including functions related with neuron growth, proliferation, axon growth and long-term potentiation.	*Curcumin derivate*	[103]
Chrysin	Amelioration of oxidative stress by reducing catalase levels, SOD, and glutathione in the cerebral cortex and hippocampus from diabetic rats. Improvement in spatial memory and learning abilities in Morris water maze test.	*Oroxylum indicum*, *Passiflora caerulea*, *Passiflora incarnata*, *Teloxys graveolens* and *Artocarpus heterophyllus*	[108]
Teasaponin	Reduction of proinflammatory citokines and inflammatory signaling in the hypothalamus from mice on high fat diet.	*Camellia sinensis*	[111]
Gallic acid	Inhibition of hippocampal neurodegeneration via its potent antioxidant and anti-inflammatory effects in diabetic rats as well as its anti-apoptotic properties.	*Phaleria macrocarpa*, *Peltiphyllum peltatum*, and *Pistacia lentiscus*	[118]
Luteolin	Neuroinflammation amelioration by reducingplasma and brain cytokines levels in a prediabetic mice. Antioxidant and anti-inflammatory effects in diabetic mice. Neuroprotection by increasing the levels of brain-derived neurotrophic factor, the action of synapsin I and postsynaptic density protein 95 in the cortex and hippocampus from mice on high fat diet. Protection against cognitive impairment in early metabolic alterations, such as prediabetic mice on a high fat diet, improvements in the Morris water maze and the step-through task.	*Salvia officinalis*, *Artemisa annua*, and others	[119,120]
Morin	Inhibition of oxidative stress and inflammation in the brain of STZ-induced diabetic rats. Neuroprotection via attenuation of ROS induced oxidative damage and neuroinflammation in experimental diabetic neuropathy.	*Maclura pomifera* and *Maclura tinctoria*	[123,124]
Rutin	Antioxidant properties in the diabetic rat retina.	*Urtica dioica* and others	[125]

**Table 2:** Natural extracts with activity at central level associated with metabolic disorders.

Natural Extract	Action	References
*Mangifera indica* Lin. extract	Reduction of spontaneous central bleeding db/db mice Restriction of microglia activation and associated inflammation in db/db mice after long-term treatment. Limitation of brain atrophy and reduction of tau hyperphosphorylation in db/db mice. Protections against learning and memory impairments in db/db mice in the Morris water maze and new object discrimination tests.	[18]
*Ficus deltoidea* leaf extract	Increased SOD and glutathione peroxidase values and reduction of thiobarbituric acid reactive substances.	[105]
*Scoparia dulcis* extract	Increase of plasma SOD, catalase or glutahione peroxidase or glutathione-S-transferase activities and reduction of gluthatione in the brain from STZ diabetic male rats. Reduction of thiobarbituric acid reactive substances and hydroperoxides formation in the brain from diabetic rats	[107]
*Gingko biloba* extract EGb 761	Scavenging reactive nitrogen and oxygen species, as well as peroxyl radicals.	[35,96,109]
Green tea extracts	Scavenging reactive nitrogen and oxygen species, as well as peroxyl radicals.	[35,110]
*Clitorea ternatea* leaf extract	Protection against oxidative stress increasing SOD, total nitric oxide, catalase and glutathione levels in the brain of diabetic rats. Improvement of spatial working memory, spatial reference memory, and spatial working-reference memory in the Y maze, the Morris water maze and radial arm maze in diabetic rats.	[112]
Grape seed extract	Beneficial effects on oxidative stress in the hippocampus of STZ-induced diabetes rats. Reduction in expression of inflammatory TNF-α, and NF-κB genes and modulation of AGEs/RAGE/NF-kappaB inflammatory pathway in the brain. Reduction of caspases 3 and 9 expression in the hippocampus, ameliorating apoptosis in diabetic rats. Improvement of cognitive impairment in diabetic rat models.	[113,114,177]
*Urtica dioica* leaves extract	Antioxidant and anti-inflamatory activities in hippocampus from STZ-induced diabetes in mice. Reduction in the number of astrocytes in the hippocampus from diabetic rats. Protection against memory deficits in different diabetic mouse models. Neuroprotective activities by iNOS downregulation, while it upregulates BDNF, TrKB, cyclin D1, Bcl2, autophagy5 and autophagy7 mRNA expression and reduces TNF-α expression in the hippocampus. Reduction of neuronal damage and DNA fragmentation. Limitation of granule cell loss of the dentate gyrus from young diabetic rats.	[115,116,117,156,157,186]
*Withania somnifera* leaf powder	Reduction of gliosis and microgliosis as well as expression of inflammation markers such as PPARγ, iNOS, MCP-1, TNF-α, IL-1β, and IL-6. Improvement of cognitive impairment STZ-treated mice, by reducing oxidative stress.	[121,184]
Extract of *Eryngium carlinae* inflorescences	Reduction of glucose levels by reducing lipid peroxidation, protein carbonylation and reactive oxigen species production, while increasing catalase activity in the brain of diabetic rats.	[122]
*Centella asiatica* extract	Protection of diabetes tissues from stress via antioxidant and anti-inflammatory mechanisms by brain reduced levels of malondialdehyde, TNF-α, IFN-γ, IL-4 or IL10.	[127]
*Ixeris gracilis* extract	Antidiabetic, antioxidant, and TNF- α lowering properties in alloxan-induced diabetic mice.	[128]
*Malvastrum tricuspidatum* extract	Restoration oxidative damage of mitochondrial status in STZ-induced diabetes.	[129]
*Astragalus Polysacharin* extract	Upregulation of phosphorylation levels of N-methyl-D-aspartate receptor, calcium/calmodulin-dependent protein kinase II and cAMP response element-binding protein, as well as reduction of the number of dead cells in the CA1 region of the hippocampus from STZ-treated diabetic rats.	[152]
*Pouteria ramiflora* extract	Hippocampal neuroprotection by restoring myosin-Va expression and the nuclear diameters of pyramidal neurons of the CA3 and the polymorphic cells of the hilus in STZ-treated rats.	[154]
*Garcinia kola* seeds	Reduced neuronal loss in regions involved in cognitive and motor functions, including the motor cortex, the medial septal nucleus a cerebellar Purkinje /granular cell layers in a T1D rat model. Improvement of cognitive abilities in diabetic rat models	[155]
*Anemarrhena rhizome* aqueous extract	Maintenance of hippocampus integrity by increasing cell proliferation and neurpeptide Y expression in the dentate gyrus from diabetic rats.	[159]
Lingonberry extract	Neuroprotective activity in diabetic rats by reducing oxidative stress and by restoring the density of purinergic receptors in the cortex.	[160]
*Ganoderma lucidum* mycelia extract	Increased resistance to apoptosis and necroptosis in T2D mice with cerebral ischemic injury.	[161]
*Andrographis paniculata* extract	Improvement of cognitive function in STZ-treated rats by reducing oxidative stress and acetylcholinesterase activity.	[175]
*Hedera nepalensis* extract	Improvement of cognitive abilities in STZ-aluminium trichloride rat model.	[176]
Kola nut extract	Protection against cognitive dysfunction in diabetic rat models.	[178]
*Brassica juncea* extract	Positive effects on learning and memory in diabetic rats.	[179]
hydroalcoholic extract of *Teucrium polium*	Limitation of cognitive impairment in the passive avoidance test and reduction of oxidative stress markers in diabetic rats.	[180]
*Rosa canina* hydro-alcoholic extract	Amelioration of cognitive impairment in mouse models after treatment.	[181]
*Ludwigia octovalvis* extract	Improvement of glycemic control and memory performance in mice fed with high fat diet. Protection against cognitive impairment in diabetic mice.	[182]
*Flos Puerariae* extract	Improvement of cognitive impairment after STZ administration, by reducing oxidative stress and restoring cholinergic activity (enhancing cholinacetyltransferase and alleviating acetylcholinesterase activities) in the cortex.	[183]
*Aloe vera* extract	Protection against cognitive impairment after STZ administration in mice, by reducing oxidative stress.	[184]
*Bacopa monnieri extracts* (CDRI-08)	Enhancement of spatial memory in T1D and T2D mice and reduction of oxidative stress.	[185]

## Abbreviations

AGEs	Advanced glycation end products
DM	Diabetes mellitus
SOD	Superoxide dismutase
STZ	Streptozotocin
TNF-α	Tumor necrosis factor α
T1D	Type 1 diabetes
T2D	Type 2 diabetes
WHO	World Health Organization

## References

[1] Cornier M.A., Dabelea D., Hernandez T.L., Lindstrom R., Steig A.J., Stob N.R., Van Pelt R.E., Wang H., Eckel R.H. (2008). The metabolic syndrome. Endocr. Rev..

[2] Craft S. (2009). The role of metabolic disorders in Alzheimer disease and vascular dementia: two roads converged. Arch Neurol..

[3] Forouhi N.G., Wareham N.J. (2014). Epidemiology of diabetes. Medicine.

[4] World Health Organization Diabetes. https://www.who.int/diabetes/en/.

[5] Skyler J.S., Bakris G.L., Bonifacio E., Darsow T., Eckel R.H., Groop L., Groop P.H., Handelsman Y., Insel R.A., Mathieu C. (2017). Differentiation of Diabetes by Pathophysiology, Natural History, and Prognosis. Diabetes.

[6] Craig M.E., Jefferies C., Dabelea D., Balde N., Seth A., Donaghue K.C. (2014). ISPAD Clinical Practice Consensus Guidelines Definition, epidemiology, and classification of diabetes in children and adolescents. Pediatr. Diabetes.

[7] Lascar N., Brown J., Pattison H., Barnett A.H., Bailey C.J., Bellary S. (2018). Type 2 diabetes in adolescents and young adults. Lancet Diabetes Endocrinol..

[8] Martin-Timon I., Sevillano-Collantes C., Segura-Galindo A., Del Canizo-Gomez F.J. (2014). Type 2 diabetes and cardiovascular disease: Have all risk factors the same strength?. World J. Diabetes.

[9] Rosenson R.S., Fioretto P., Dodson P.M. (2011). Does microvascular disease predict macrovascular events in type 2 diabetes?. Atherosclerosis.

[10] Craft S. (2012). Alzheimer disease: Insulin resistance and AD--extending the translational path. Nat. Rev. Neurol..

[11] Strachan M.W., Reynolds R.M., Frier B.M., Mitchell R.J., Price J.F. (2009). The role of metabolic derangements and glucocorticoid excess in the aetiology of cognitive impairment in type 2 diabetes. Implications for future therapeutic strategies. Diabetes Obesity Metab..

[12] Hamed S.A. (2017). Brain injury with diabetes mellitus: evidence, mechanisms and treatment implications. Expert Rev. Clin. Pharmacol..

[13] Kodl C.T., Franc D.T., Rao J.P., Anderson F.S., Thomas W., Mueller B.A., Lim K.O., Seaquist E.R. (2008). Diffusion tensor imaging identifies deficits in white matter microstructure in subjects with type 1 diabetes that correlate with reduced neurocognitive function. Diabetes.

[14] Ryan C.M., Geckle M.O., Orchard T.J. (2003). Cognitive efficiency declines over time in adults with Type 1 diabetes: effects of micro- and macrovascular complications. Diabetologia.

[15] Moran C., Beare R., Phan T.G., Bruce D.G., Callisaya M.L., Srikanth V., Alzheimer’s Disease Neuroimaging Initiative (ADNI) (2015). Type 2 diabetes mellitus and biomarkers of neurodegeneration. Neurology.

[16] Fishel M.A., Watson G.S., Montine T.J., Wang Q., Green P.S., Kulstad J.J., Cook D.G., Peskind E.R., Baker L.D., Goldgaber D. (2005). Hyperinsulinemia provokes synchronous increases in central inflammation and beta-amyloid in normal adults. Arch. Neurol..

[17] Wang T., Fu F., Han B., Zhang L., Zhang X. (2012). Danshensu ameliorates the cognitive decline in streptozotocin-induced diabetic mice by attenuating advanced glycation end product-mediated neuroinflammation. J. Neuroimmunol..

[18] Infante-Garcia C., Jose Ramos-Rodriguez J., Marin-Zambrana Y., Teresa Fernandez-Ponce M., Casas L., Mantell C., Garcia-Alloza M. (2017). Mango leaf extract improves central pathology and cognitive impairment in a type 2 diabetes mouse model. Brain Pathol..

[19] Moran C., Beare R., Wang W., Callisaya M., Srikanth V., Alzheimer’s Disease Neuroimaging Initiative (ADNI) (2019). Type 2 diabetes mellitus, brain atrophy, and cognitive decline. Neurology.

[20] Luchsinger J.A., Reitz C., Honig L.S., Tang M.X., Shea S., Mayeux R. (2005). Aggregation of vascular risk factors and risk of incident Alzheimer disease. Neurology.

[21] Luchsinger J.A., Tang M.X., Shea S., Mayeux R. (2004). Hyperinsulinemia and risk of Alzheimer disease. Neurology.

[22] Matsuzaki T., Sasaki K., Tanizaki Y., Hata J., Fujimi K., Matsui Y., Sekita A., Suzuki S.O., Kanba S., Kiyohara Y. (2010). Insulin resistance is associated with the pathology of Alzheimer disease: the Hisayama study. Neurology.

[23] Schrijvers E.M., Witteman J.C., Sijbrands E.J., Hofman A., Koudstaal P.J., Breteler M.M. (2010). Insulin metabolism and the risk of Alzheimer disease: the Rotterdam Study. Neurology.

[24] Strachan M.W., Reynolds R.M., Frier B.M., Mitchell R.J., Price J.F. (2008). The relationship between type 2 diabetes and dementia. Br. Med. Bull..

[25] Newman D.J., Cragg G.M. (2016). Natural Products as Sources of New Drugs from 1981 to 2014. J. Nat. Prod..

[26] Flores-Jimenez N.G., Rojas-Lemus M., Fortoul T.I., Zepeda-Rodriguez A., Lopez-Camacho P.Y., Anacleto-Santos J., Malagon-Gutierrez F., Basurto-Islas G., Rivera-Fernandez N. (2018). Histopathological alterations in mice under sub-acute treatment with Hintonia latiflora methanolic stem bark extract. Histol. Histopathol..

[27] Spagnuolo C., Napolitano M., Tedesco I., Moccia S., Milito A., Russo G.L. (2016). Neuroprotective Role of Natural Polyphenols. Curr. Top. Med. Chem..

[28] Cheynier V., Comte G., Davies K.M., Lattanzio V., Martens S. (2013). Plant phenolics: recent advances on their biosynthesis, genetics, and ecophysiology. Plant Physiol. Biochem..

[29] Sevastre-Berghian A.C., Toma V.A., Sevastre B., Hanganu D., Vlase L., Benedec D., Oniga I., Baldea I., Olteanu D., Moldovan R. (2018). Characterization and biological effects of Hypericum extracts on experimentally-induced - anxiety, oxidative stress and inflammation in rats. J. Physiol. Pharmacol..

[30] Spagnuolo C., Moccia S., Russo G.L. (2018). Anti-inflammatory effects of flavonoids in neurodegenerative disorders. Eur. J. Med. Chem..

[31] Lima M.C., Paiva de Sousa C., Fernandez-Prada C., Harel J., Dubreuil J.D., de Souza E.L. (2019). A review of the current evidence of fruit phenolic compounds as potential antimicrobials against pathogenic bacteria. Microb. Pathog..

[32] Christman L.M., Dean L.L., Allen J.C., Godinez S.F., Toomer O.T. (2019). Peanut skin phenolic extract attenuates hyperglycemic responses in vivo and in vitro. PloS ONE.

[33] Pohl F., Kong Thoo Lin P. (2018). The Potential Use of Plant Natural Products and Plant Extracts with Antioxidant Properties for the Prevention/Treatment of Neurodegenerative Diseases: In Vitro, In Vivo and Clinical Trials. Molecules.

[34] Infante-Garcia C., Ramos-Rodriguez J.J., Delgado-Olmos I., Gamero-Carrasco C., Fernandez-Ponce M.T., Casas L., Mantell C., Garcia-Alloza M. (2017). Long-Term Mangiferin Extract Treatment Improves Central Pathology and Cognitive Deficits in APP/PS1 Mice. Mol. Neurobiol..

[35] Figueira I., Menezes R., Macedo D., Costa I., Dos Santos C.N. (2017). Polyphenols Beyond Barriers: A Glimpse into the Brain. Curr. Neuropharmacol..

[36] Tsao R. (2010). Chemistry and biochemistry of dietary polyphenols. Nutrients.

[37] Garcia-Alloza M., Dodwell S.A., Meyer-Luehmann M., Hyman B.T., Bacskai B.J. (2006). Plaque-derived oxidative stress mediates distorted neurite trajectories in the Alzheimer mouse model. J. Neuropathol. Exp. Neurol..

[38] Liu Y.W., Liu X.L., Kong L., Zhang M.Y., Chen Y.J., Zhu X., Hao Y.C. (2019). Neuroprotection of quercetin on central neurons against chronic high glucose through enhancement of Nrf2/ARE/glyoxalase-1 pathway mediated by phosphorylation regulation. Biomed. Pharmacother..

[39] Fu Q.Y., Li Q.S., Lin X.M., Qiao R.Y., Yang R., Li X.M., Dong Z.B., Xiang L.P., Zheng X.Q., Lu J.L. (2017). Antidiabetic Effects of Tea. Molecules.

[40] Dominguez Avila J.A., Rodrigo Garcia J., Gonzalez Aguilar G.A., de la Rosa L.A. (2017). The Antidiabetic Mechanisms of Polyphenols Related to Increased Glucagon-Like Peptide-1 (GLP1) and Insulin Signaling. Molecules.

[41] Serna-Thome G., Castro-Eguiluz D., Fuchs-Tarlovsky V., Sanchez-Lopez M., Delgado-Olivares L., Coronel-Martinez J., Molina-Trinidad E.M., de la Torre M., Cetina-Perez L. (2018). Use of Functional Foods and Oral Supplements as Adjuvants in Cancer Treatment. Rev. Inves. Clin..

[42] Biessels G.J., Staekenborg S., Brunner E., Brayne C., Scheltens P. (2006). Risk of dementia in diabetes mellitus: A systematic review. Lancet Neurol..

[43] Crane P.K., Walker R., Hubbard R.A., Li G., Nathan D.M., Zheng H., Haneuse S., Craft S., Montine T.J., Kahn S.E. (2013). Glucose levels and risk of dementia. N. Engl. J. Med..

[44] Infante-Garcia C., Ramos-Rodriguez J.J., Hierro-Bujalance C., Ortegon E., Pickett E., Jackson R., Hernandez-Pacho F., Spires-Jones T., Garcia-Alloza M. (2018). Antidiabetic Polypill Improves Central Pathology and Cognitive Impairment in a Mixed Model of Alzheimer’s Disease and Type 2 Diabetes. Mol. Neurobiol..

[45] Munhoz A.C.M., Frode T.S. (2018). Isolated Compounds from Natural Products with Potential Antidiabetic Activity - A Systematic Review. Curr. Diabetes Rev..

[46] Chen T.Y., Ferruzzi M.G., Wu Q.L., Simon J.E., Talcott S.T., Wang J., Ho L., Todd G., Cooper B., Pasinetti G.M. (2017). Influence of diabetes on plasma pharmacokinetics and brain bioavailability of grape polyphenols and their phase II metabolites in the Zucker diabetic fatty rat. Mol. Nutr. Food Res..

[47] Domingueti C.P., Dusse L.M., Carvalho M., de Sousa L.P., Gomes K.B., Fernandes A.P. (2016). Diabetes mellitus: The linkage between oxidative stress, inflammation, hypercoagulability and vascular complications. J. Diabetes Complicat..

[48] Goldberg R.B. (2009). Cytokine and cytokine-like inflammation markers, endothelial dysfunction, and imbalanced coagulation in development of diabetes and its complications. J. Clin. Endocrinol. Metab..

[49] Wautier J.L., Guillausseau P.J. (1998). Diabetes, advanced glycation endproducts and vascular disease. Vasc. Med..

[50] Reddy G.K. (2004). AGE-related cross-linking of collagen is associated with aortic wall matrix stiffness in the pathogenesis of drug-induced diabetes in rats. Microvasc. Res..

[51] Idris I., Thomson G.A., Sharma J.C. (2006). Diabetes mellitus and stroke. Int. J. Clin. Pract..

[52] Callahan A., Amarenco P., Goldstein L.B., Sillesen H., Messig M., Samsa G.P., Altafullah I., Ledbetter L.Y., MacLeod M.J., Scott R. (2011). Risk of stroke and cardiovascular events after ischemic stroke or transient ischemic attack in patients with type 2 diabetes or metabolic syndrome: secondary analysis of the Stroke Prevention by Aggressive Reduction in Cholesterol Levels (SPARCL) trial. Arch. Neurol..

[53] Mogi M., Horiuchi M. (2011). Neurovascular coupling in cognitive impairment associated with diabetes mellitus. Circ. J..

[54] Hayden M.R., Grant D.G., Aroor A.R., DeMarco V.G. (2019). Empagliflozin Ameliorates Type 2 Diabetes-Induced Ultrastructural Remodeling of the Neurovascular Unit and Neuroglia in the Female db/db Mouse. Brain Sci..

[55] Infante-Garcia C., Ramos-Rodriguez J.J., Galindo-Gonzalez L., Garcia-Alloza M. (2016). Long-term central pathology and cognitive impairment are exacerbated in a mixed model of Alzheimer’s disease and type 2 diabetes. Psychoneuroendocrinology.

[56] Ramos-Rodriguez J.J., Jimenez-Palomares M., Murillo-Carretero M.I., Infante-Garcia C., Berrocoso E., Hernandez-Pacho F., Lechuga-Sancho A.M., Cozar-Castellano I., Garcia-Alloza M. (2015). Central vascular disease and exacerbated pathology in a mixed model of type 2 diabetes and Alzheimer’s disease. Psychoneuroendocrinology.

[57] Zhang L., Chopp M., Zhang Y., Xiong Y., Li C., Sadry N., Rhaleb I., Lu M., Zhang Z.G. (2016). Diabetes Mellitus Impairs Cognitive Function in Middle-Aged Rats and Neurological Recovery in Middle-Aged Rats After Stroke. Stroke.

[58] Pasquier F., Boulogne A., Leys D., Fontaine P. (2006). Diabetes mellitus and dementia. Diabetes Metab..

[59] Wang S., Cao C., Chen Z., Bankaitis V., Tzima E., Sheibani N., Burridge K. (2012). Pericytes regulate vascular basement membrane remodeling and govern neutrophil extravasation during inflammation. PloS ONE.

[60] Bogush M., Heldt N.A., Persidsky Y. (2017). Blood Brain Barrier Injury in Diabetes: Unrecognized Effects on Brain and Cognition. J. Neuroimmune Pharmacol..

[61] Manasson J., Tien T., Moore C., Kumar N.M., Roy S. (2013). High glucose-induced downregulation of connexin 30.2 promotes retinal vascular lesions: implications for diabetic retinopathy. Investig. Ophthalmol. Vis. Sci..

[62] Sajja R.K., Prasad S., Cucullo L. (2014). Impact of altered glycaemia on blood-brain barrier endothelium: an in vitro study using the hCMEC/D3 cell line. Fluids Barriers CNS..

[63] Li B., Li Y., Liu K., Wang X., Qi J., Wang B., Wang Y. (2017). High glucose decreases claudins-5 and -11 in cardiac microvascular endothelial cells: Antagonistic effects of tongxinluo. Endocr. Res..

[64] Maile L.A., Gollahon K., Wai C., Dunbar P., Busby W., Clemmons D. (2014). Blocking alphaVbeta3 integrin ligand occupancy inhibits the progression of albuminuria in diabetic rats. J. Diabetes Res..

[65] Park S.W., Yun J.H., Kim J.H., Kim K.W., Cho C.H., Kim J.H. (2014). Angiopoietin 2 induces pericyte apoptosis via alpha3beta1 integrin signaling in diabetic retinopathy. Diabetes.

[66] Lee Y.J., Jung S.H., Kim S.H., Kim M.S., Lee S., Hwang J., Kim S.Y., Kim Y.M., Ha K.S. (2016). Essential Role of Transglutaminase 2 in Vascular Endothelial Growth Factor-Induced Vascular Leakage in the Retina of Diabetic Mice. Diabetes.

[67] Abu El-Asrar A.M., Mohammad G., Nawaz M.I., Abdelsaid M., Siddiquei M.M., Alam K., Van den Eynde K., De Hertogh G., Opdenakker G., Al-Shabrawey M. (2015). The Chemokine Platelet Factor-4 Variant (PF-4var)/CXCL4L1 Inhibits Diabetes-Induced Blood-Retinal Barrier Breakdown. Investig. Ophthalmol. Vis. Sci..

[68] Price T.O., Eranki V., Banks W.A., Ercal N., Shah G.N. (2012). Topiramate treatment protects blood-brain barrier pericytes from hyperglycemia-induced oxidative damage in diabetic mice. Endocrinology.

[69] Takechi R., Lam V., Brook E., Giles C., Fimognari N., Mooranian A., Al-Salami H., Coulson S.H., Nesbit M., Mamo J.C.L. (2017). Blood-Brain Barrier Dysfunction Precedes Cognitive Decline and Neurodegeneration in Diabetic Insulin Resistant Mouse Model: An Implication for Causal Link. Front. Aging Neurosci..

[70] Ramos-Rodriguez J.J., Ortiz O., Jimenez-Palomares M., Kay K.R., Berrocoso E., Murillo-Carretero M.I., Perdomo G., Spires-Jones T., Cozar-Castellano I., Lechuga-Sancho A.M. (2013). Differential central pathology and cognitive impairment in pre-diabetic and diabetic mice. Psychoneuroendocrinology.

[71] Jin Y., Khadka D.B., Cho W.J. (2016). Pharmacological effects of berberine and its derivatives: A patent update. Expert Opin. Ther. Pat..

[72] Yin J., Xing H., Ye J. (2008). Efficacy of berberine in patients with type 2 diabetes mellitus. Metabolism.

[73] Yin S., Bai W., Li P., Jian X., Shan T., Tang Z., Jing X., Ping S., Li Q., Miao Z. (2018). Berberine suppresses the ectopic expression of miR-133a in endothelial cells to improve vascular dementia in diabetic rats. Clin. Exp. Hypertens..

[74] Wei L.L., Chen Y., Yu Q.Y., Wang Y., Liu G. (2018). Patchouli alcohol protects against ischemia/reperfusion-induced brain injury via inhibiting neuroinflammation in normal and obese mice. Brain Res..

[75] Patel S.S., Udayabanu M. (2017). Effect of natural products on diabetes associated neurological disorders. Rev. Neurosci..

[76] Wong R.H., Raederstorff D., Howe P.R. (2016). Acute Resveratrol Consumption Improves Neurovascular Coupling Capacity in Adults with Type 2 Diabetes Mellitus. Nutrients.

[77] Jing Y.H., Chen K.H., Kuo P.C., Pao C.C., Chen J.K. (2013). Neurodegeneration in streptozotocin-induced diabetic rats is attenuated by treatment with resveratrol. Neuroendocrinology.

[78] Newcombe E.A., Camats-Perna J., Silva M.L., Valmas N., Huat T.J., Medeiros R. (2018). Inflammation: The link between comorbidities, genetics, and Alzheimer’s disease. J. Neuroinflammation.

[79] Hotamisligil G.S. (2006). Inflammation and metabolic disorders. Nature.

[80] Hotamisligil G.S., Shargill N.S., Spiegelman B.M. (1993). Adipose expression of tumor necrosis factor-alpha: Direct role in obesity-linked insulin resistance. Science.

[81] Wellen K.E., Hotamisligil G.S. (2005). Inflammation, stress, and diabetes. J. Clin. Investig..

[82] Colombo E., Farina C. (2016). Astrocytes: Key Regulators of Neuroinflammation. Trends Immunol..

[83] Jeong H.K., Ji K., Min K., Joe E.H. (2013). Brain inflammation and microglia: Facts and misconceptions. Exp. Neurobiol..

[84] Ransohoff R.M., Engelhardt B. (2012). The anatomical and cellular basis of immune surveillance in the central nervous system. Nat. Rev. Immunol..

[85] Ferreira R., Bernardino L. (2015). Dual role of microglia in health and disease: pushing the balance toward repair. Front Cell Neurosci..

[86] Chen Z., Trapp B.D. (2016). Microglia and neuroprotection. J. Neurochem..

[87] Ramos-Rodriguez J.J., Ortiz-Barajas O., Gamero-Carrasco C., de la Rosa P.R., Infante-Garcia C., Zopeque-Garcia N., Lechuga-Sancho A.M., Garcia-Alloza M. (2014). Prediabetes-induced vascular alterations exacerbate central pathology in APPswe/PS1dE9 mice. Psychoneuroendocrinology.

[88] Ramos-Rodriguez J.J., Infante-Garcia C., Galindo-Gonzalez L., Garcia-Molina Y., Lechuga-Sancho A., Garcia-Alloza M. (2016). Increased Spontaneous Central Bleeding and Cognition Impairment in APP/PS1 Mice with Poorly Controlled Diabetes Mellitus. Mol. Neurobiol..

[89] Ramos-Rodriguez J.J., Sanchez-Sotano D., Doblas-Marquez A., Infante-Garcia C., Lubian-Lopez S., Garcia-Alloza M. (2017). Intranasal insulin reverts central pathology and cognitive impairment in diabetic mother offspring. Mol. Neurodegener..

[90] Van Dyken P., Lacoste B. (2018). Impact of Metabolic Syndrome on Neuroinflammation and the Blood-Brain Barrier. Front. Neurosci..

[91] Hwang I.K., Choi J.H., Nam S.M., Park O.K., Yoo D.Y., Kim W., Yi S.S., Won M.H., Seong J.K., Yoon Y.S. (2014). Activation of microglia and induction of pro-inflammatory cytokines in the hippocampus of type 2 diabetic rats. Neurol. Res..

[92] Ibrahim A.S., El-Shishtawy M.M., Pena A., Liou G.I. (2010). Genistein attenuates retinal inflammation associated with diabetes by targeting of microglial activation. Mol. Vis..

[93] Lauridsen J.K., Olesen R.H., Vendelbo J., Hyde T.M., Kleinman J.E., Bibby B.M., Brock B., Rungby J., Larsen A. (2017). High BMI levels associate with reduced mRNA expression of IL10 and increased mRNA expression of iNOS (NOS2) in human frontal cortex. Transl. Psychiatry.

[94] Chen W., Jia Z., Pan M.-H., Babu P.V.A. (2016). Natural Products for the Prevention of Oxidative Stress-Related Diseases: Mechanisms and Strategies. Oxidative Med. Cell. Longev..

[95] Jia Z., Babu P.V.A., Chen W., Sun X. (2018). Natural Products Targeting on Oxidative Stress and Inflammation: Mechanisms, Therapies, and Safety Assessment. Oxidative Med. Cell. Longev..

[96] Maitra I., Marcocci L., Droy-Lefaix M.T., Packer L. (1995). Peroxyl radical scavenging activity of Ginkgo biloba extract EGb 761. Biochem. Pharmacol..

[97] Frandsen J.R., Narayanasamy P. (2018). Neuroprotection through flavonoid: Enhancement of the glyoxalase pathway. Redox Biol..

[98] Mahesh T., Menon V.P. (2004). Quercetin allievates oxidative stress in streptozotocin-induced diabetic rats. Phytother. Res..

[99] Bournival J., Francoeur M.A., Renaud J., Martinoli M.G. (2012). Quercetin and sesamin protect neuronal PC12 cells from high-glucose-induced oxidation, nitrosative stress, and apoptosis. Rejuvenation Res..

[100] Marquez L., Garcia-Bueno B., Madrigal J.L., Leza J.C. (2012). Mangiferin decreases inflammation and oxidative damage in rat brain after stress. Eur. J. Nutr..

[101] Liu Y.W., Cheng Y.Q., Liu X.L., Hao Y.C., Li Y., Zhu X., Zhang F., Yin X.X. (2017). Mangiferin Upregulates Glyoxalase 1 Through Activation of Nrf2/ARE Signaling in Central Neurons Cultured with High Glucose. Mol. Neurobiol..

[102] Faheem N.M., El Askary A. (2017). Neuroprotective role of curcumin on the hippocampus against the structural and serological alterations of streptozotocin-induced diabetes in Sprague Dawely rats. Iran. J. Basic Med. Sci..

[103] Daugherty D.J., Marquez A., Calcutt N.A., Schubert D. (2018). A novel curcumin derivative for the treatment of diabetic neuropathy. Neuropharmacology.

[104] Sadi G., Konat D. (2016). Resveratrol regulates oxidative biomarkers and antioxidant enzymes in the brain of streptozotocin-induced diabetic rats. Pharm. Biol..

[105] Nurdiana S., Goh Y.M., Hafandi A., Dom S.M., Nur Syimal’ain A., Noor Syaffinaz N.M., Ebrahimi M. (2018). Improvement of spatial learning and memory, cortical gyrification patterns and brain oxidative stress markers in diabetic rats treated with Ficus deltoidea leaf extract and vitexin. J. Tradit. Complement. Med..

[106] Samarghandian S., Azimi-Nezhad M., Samini F. (2014). Ameliorative Effect of Saffron Aqueous Extract on Hyperglycemia, Hyperlipidemia, and Oxidative Stress on Diabetic Encephalopathy in Streptozotocin Induced Experimental Diabetes Mellitus. BioMed Int..

[107] Pari L., Latha M. (2004). Protective role of Scoparia dulcis plant extract on brain antioxidant status and lipidperoxidation in STZ diabetic male Wistar rats. BMC Complement Altern Med..

[108] Li R., Zang A., Zhang L., Zhang H., Zhao L., Qi Z., Wang H. (2014). Chrysin ameliorates diabetes-associated cognitive deficits in Wistar rats. Neurol. Sci..

[109] Marcocci L., Packer L., Droy-Lefaix M.T., Sekaki A., Gardes-Albert M. (1994). Antioxidant action of Ginkgo biloba extract EGb. Methods Enzymol..

[110] Choi H.R., Choi J.S., Han Y.N., Bae S.J., Chung H.Y. (2002). Peroxynitrite scavenging activity of herb extracts. Phytother. Res..

[111] Yu Y., Wu Y., Szabo A., Wu Z., Wang H., Li D., Huang X.F. (2013). Teasaponin reduces inflammation and central leptin resistance in diet-induced obese male mice. Endocrinology.

[112] Talpate K.A., Bhosale U.A., Zambare M.R., Somani R.S. (2014). Neuroprotective and nootropic activity of Clitorea ternatea Linn.(Fabaceae) leaves on diabetes induced cognitive decline in experimental animals. J. Pharm. Bioallied Sci..

[113] Yonguc G.N., Dodurga Y., Adiguzel E., Gundogdu G., Kucukatay V., Ozbal S., Yilmaz I., Cankurt U., Yilmaz Y., Akdogan I. (2015). Grape seed extract has superior beneficial effects than vitamin E on oxidative stress and apoptosis in the hippocampus of streptozotocin induced diabetic rats. Gene.

[114] Lu M., Xu L., Li B., Zhang W., Zhang C., Feng H., Cui X., Gao H. (2010). Protective effects of grape seed proanthocyanidin extracts on cerebral cortex of streptozotocin-induced diabetic rats through modulating AGEs/RAGE/NF-kappaB pathway. J. Nutr. Sci. Vitaminol..

[115] Patel S.S., Gupta S., Udayabanu M. (2016). Urtica dioica modulates hippocampal insulin signaling and recognition memory deficit in streptozotocin induced diabetic mice. Metab. Brain Dis..

[116] Patel S.S., Parashar A., Udayabanu M. (2015). Urtica dioica leaves modulates muscarinic cholinergic system in the hippocampus of streptozotocin-induced diabetic mice. Metab. Brain Dis..

[117] Jahanshahi M., Golalipour M.J., Afshar M. (2009). The effect of Urtica dioica extract on the number of astrocytes in the dentate gyrus of diabetic rats. Folia Morphol..

[118] Abdel-Moneim A., Yousef A.I., Abd El-Twab S.M., Abdel Reheim E.S., Ashour M.B. (2017). Gallic acid and p-coumaric acid attenuate type 2 diabetes-induced neurodegeneration in rats. Metab. Brain Dis..

[119] Liu Y., Fu X., Lan N., Li S., Zhang J., Wang S., Li C., Shang Y., Huang T., Zhang L. (2014). Luteolin protects against high fat diet-induced cognitive deficits in obesity mice. Behav. Brain Res..

[120] Liu Y., Tian X., Gou L., Sun L., Ling X., Yin X. (2013). Luteolin attenuates diabetes-associated cognitive decline in rats. Brain Res. Bull..

[121] Kaur T., Kaur G. (2017). Withania somnifera as a potential candidate to ameliorate high fat diet-induced anxiety and neuroinflammation. J. Neuroinflammation.

[122] Pena-Montes D.J., Huerta-Cervantes M., Rios-Silva M., Trujillo X., Huerta M., Noriega-Cisneros R., Salgado-Garciglia R., Saavedra-Molina A. (2019). Protective Effect of the Hexanic Extract of Eryngium carlinae Inflorescences In Vitro, in Yeast, and in Streptozotocin-Induced Diabetic Male Rats. Antioxidants.

[123] Ola M.S., Aleisa A.M., Al-Rejaie S.S., Abuohashish H.M., Parmar M.Y., Alhomida A.S., Ahmed M.M. (2014). Flavonoid, morin inhibits oxidative stress, inflammation and enhances neurotrophic support in the brain of streptozotocin-induced diabetic rats. Neurol. Sci..

[124] Bachewal P., Gundu C., Yerra V.G., Kalvala A.K., Areti A., Kumar A. (2018). Morin exerts neuroprotection via attenuation of ROS induced oxidative damage and neuroinflammation in experimental diabetic neuropathy. BioFactors.

[125] Ola M.S., Ahmed M.M., Ahmad R., Abuohashish H.M., Al-Rejaie S.S., Alhomida A.S. (2015). Neuroprotective Effects of Rutin in Streptozotocin-Induced Diabetic Rat Retina. J. Mol. Neurosci..

[126] Moghaddam H.K., Baluchnejadmojarad T., Roghani M., Khaksari M., Norouzi P., Ahooie M., Mahboobi F. (2014). Berberine ameliorate oxidative stress and astrogliosis in the hippocampus of STZ-induced diabetic rats. Mol. Neurobiol..

[127] Masola B., Oguntibeju O.O., Oyenihi A.B. (2018). Centella asiatica ameliorates diabetes-induced stress in rat tissues via influences on antioxidants and inflammatory cytokines. Biomed. Pharmacother..

[128] Syiem D., Warjri P. (2015). Antidiabetic, antioxidant, and TNF-alpha lowering properties of extract of the traditionally used plant Ixeris gracilis in alloxan-induced diabetic mice. Pharmaceutical Biol..

[129] Solanki I., Parihar P., Shetty R., Parihar M.S. (2017). Synaptosomal and mitochondrial oxidative damage followed by behavioral impairments in streptozotocin induced diabetes mellitus: restoration by Malvastrum tricuspidatum. Cell Mol. Biol..

[130] van Harten B., de Leeuw F.E., Weinstein H.C., Scheltens P., Biessels G.J. (2006). Brain imaging in patients with diabetes: a systematic review. Diabetes Care.

[131] Marseglia A., Fratiglioni L., Kalpouzos G., Wang R., Backman L., Xu W. (2019). Prediabetes and diabetes accelerate cognitive decline and predict microvascular lesions: A population-based cohort study. Alzheimer’s Dement..

[132] Ramos-Rodriguez J.J., Spires-Jones T., Pooler A.M., Lechuga-Sancho A.M., Bacskai B.J., Garcia-Alloza M. (2017). Progressive Neuronal Pathology and Synaptic Loss Induced by Prediabetes and Type 2 Diabetes in a Mouse Model of Alzheimer’s Disease. Mol. Neurobiol..

[133] Moran C., Tapp R.J., Hughes A.D., Magnussen C.G., Blizzard L., Phan T.G., Beare R., Witt N., Venn A., Munch G. (2016). The Association of Type 2 Diabetes Mellitus with Cerebral Gray Matter Volume Is Independent of Retinal Vascular Architecture and Retinopathy. J. Diabetes Res..

[134] Bednarik P., Moheet A.A., Grohn H., Kumar A.F., Eberly L.E., Seaquist E.R., Mangia S. (2017). Type 1 Diabetes and Impaired Awareness of Hypoglycemia Are Associated with Reduced Brain Gray Matter Volumes. Front. Neurosci..

[135] McCrimmon R.J., Ryan C.M., Frier B.M. (2012). Diabetes and cognitive dysfunction. Lancet.

[136] Kumar A., Haroon E., Darwin C., Pham D., Ajilore O., Rodriguez G., Mintz J. (2008). Gray matter prefrontal changes in type 2 diabetes detected using MRI. J. Magn. Reson. Imaging: Jmri..

[137] de Bresser J., Tiehuis A.M., van den Berg E., Reijmer Y.D., Jongen C., Kappelle L.J., Mali W.P., Viergever M.A., Biessels G.J., Utrecht Diabetic Encephalopathy Study Group (2010). Progression of cerebral atrophy and white matter hyperintensities in patients with type 2 diabetes. Diabetes Care.

[138] Convit A., Wolf O.T., Tarshish C., de Leon M.J. (2003). Reduced glucose tolerance is associated with poor memory performance and hippocampal atrophy among normal elderly. Proc. Natl. Acad. Sci. USA.

[139] Muriach M., Flores-Bellver M., Romero F.J., Barcia J.M. (2014). Diabetes and the brain: oxidative stress, inflammation, and autophagy. Oxidative Med. Cell. Longev..

[140] Zhao W.Q., Alkon D.L. (2001). Role of insulin and insulin receptor in learning and memory. Mol. Cell. Endocrinol..

[141] Beckman K.B., Ames B.N. (1998). The free radical theory of aging matures. Physiol. Rev..

[142] Merad-Boudia M., Nicole A., Santiard-Baron D., Saille C., Ceballos-Picot I. (1998). Mitochondrial impairment as an early event in the process of apoptosis induced by glutathione depletion in neuronal cells: relevance to Parkinson’s disease. Biochem. Pharmacol..

[143] Bharadwaj P., Wijesekara N., Liyanapathirana M., Newsholme P., Ittner L., Fraser P., Verdile G. (2017). The Link between Type 2 Diabetes and Neurodegeneration: Roles for Amyloid-beta, Amylin, and Tau Proteins. J. Alzheimer’s Dis..

[144] Kimura Y., Ito H., Ohnishi R., Hatano T. (2010). Inhibitory effects of polyphenols on human cytochrome P450 3A4 and 2C9 activity. Food Chem. Toxicol..

[145] Espargaro A., Ginex T., Vadell M.D., Busquets M.A., Estelrich J., Munoz-Torrero D., Luque F.J., Sabate R. (2017). Combined in Vitro Cell-Based/in Silico Screening of Naturally Occurring Flavonoids and Phenolic Compounds as Potential Anti-Alzheimer Drugs. J. Nat. Products..

[146] Cittadini M.C., Repossi G., Albrecht C., Di Paola Naranjo R., Miranda A.R., de Pascual-Teresa S., Soria E.A. (2019). Effects of bioavailable phenolic compounds from Ilex paraguariensis on the brain of mice with lung adenocarcinoma. Phytother. Res..

[147] Chan E.W.L., Yeo E.T.Y., Wong K.W.L., See M.L., Wong K.Y., Gan S.Y. (2019). Piper sarmentosum Roxb. Root Extracts Confer Neuroprotection by Attenuating Beta Amyloid-Induced Pro-Inflammatory Cytokines Released from Microglial Cells. Curr. Alzheimer Res..

[148] Kean R.J., Lamport D.J., Dodd G.F., Freeman J.E., Williams C.M., Ellis J.A., Butler L.T., Spencer J.P. (2015). Chronic consumption of flavanone-rich orange juice is associated with cognitive benefits: an 8-wk, randomized, double-blind, placebo-controlled trial in healthy older adults. Am. J. Clin. Nutrition..

[149] Vauzour D. (2012). Dietary polyphenols as modulators of brain functions: biological actions and molecular mechanisms underpinning their beneficial effects. Oxidative Med. Cell. Longev..

[150] Tian X., Liu Y., Ren G., Yin L., Liang X., Geng T., Dang H., An R. (2016). Resveratrol limits diabetes-associated cognitive decline in rats by preventing oxidative stress and inflammation and modulating hippocampal structural synaptic plasticity. Brain Res..

[151] Tian Z., Wang J., Xu M., Wang Y., Zhang M., Zhou Y. (2016). Resveratrol Improves Cognitive Impairment by Regulating Apoptosis and Synaptic Plasticity in Streptozotocin-Induced Diabetic Rats. Cell. Physiol. Biochem..

[152] Zhang G., Fang H., Li Y., Xu J., Zhang D., Sun Y., Zhou L., Zhang H. (2019). Neuroprotective Effect of Astragalus Polysacharin on Streptozotocin (STZ)-Induced Diabetic Rats. Med. Sci. Monit..

[153] Matysek M., Mozel S., Szalak R., Zacharko-Siembida A., Obszanska K., Arciszewski M.B. (2017). Effect of feeding with bilberry fruit on the expression pattern of alphaCaMKII in hippocampal neurons in normal and diabetic rats. Polish J. Vet. Sci..

[154] da Costa A.V., Calabria L.K., Furtado F.B., de Gouveia N.M., Oliveira R.J., de Oliveira V.N., Beletti M.E., Espindola F.S. (2013). Neuroprotective effects of Pouteria ramiflora (Mart.) Radlk (Sapotaceae) extract on the brains of rats with streptozotocin-induced diabetes. Metab. Brain Dis..

[155] Seke Etet P.F., Farahna M., Satti G.M.H., Bushara Y.M., El-Tahir A., Hamza M.A., Osman S.Y., Dibia A.C., Vecchio L. (2017). Garcinia kola seeds may prevent cognitive and motor dysfunctions in a type 1 diabetes mellitus rat model partly by mitigating neuroinflammation. J. Complement Integr. Med..

[156] Patel S.S., Ray R.S., Sharma A., Mehta V., Katyal A., Udayabanu M. (2018). Antidepressant and anxiolytic like effects of Urtica dioica leaves in streptozotocin induced diabetic mice. Metab. Brain.

[157] Fazeli S.A., Gharravi A.M., Ghafari S., Jahanshahi M., Golalipour M.J. (2008). The granule cell density of the dentate gyrus following administration of Urtica dioica extract to young diabetic rats. Folia Morphol..

[158] Molahosseini A., Taghavi M.M., Taghipour Z., Shabanizadeh A., Fatehi F., Kazemi Arababadi M., Eftekhar Vaghefe S.H. (2016). The effect of the ginger on the apoptosis of hippochampal cells according to the expression of BAX and Cyclin D1 genes and histological characteristics of brain in streptozotocin male diabetic rats. Cell. Mol. Biol..

[159] Shin M.S., Kim S.K., Kim Y.S., Kim S.E., Ko I.G., Kim C.J., Kim Y.M., Kim B.K., Kim T.S. (2008). Aqueous extract of Anemarrhena rhizome increases cell proliferation and neuropeptide Y expression in the hippocampal dentate gyrus on streptozotocin-induced diabetic rats. Fitoterapia.

[160] Reichert K.P., Schetinger M.R.C., Gutierres J.M., Pelinson L.P., Stefanello N., Dalenogare D.P., Baldissarelli J., Lopes T.F., Morsch V.M. (2018). Lingonberry Extract Provides Neuroprotection by Regulating the Purinergic System and Reducing Oxidative Stress in Diabetic Rats. Mol. Nutr. Food Res..

[161] Xuan M., Okazaki M., Iwata N., Asano S., Kamiuchi S., Matsuzaki H., Sakamoto T., Miyano Y., Iizuka H., Hibino Y. (2015). Chronic Treatment with a Water-Soluble Extract from the Culture Medium of Ganoderma lucidum Mycelia Prevents Apoptosis and Necroptosis in Hypoxia/Ischemia-Induced Injury of Type 2 Diabetic Mouse Brain. Evid Based Complement Alternat. Med..

[162] Koekkoek P.S., Kappelle L.J., van den Berg E., Rutten G.E., Biessels G.J. (2015). Cognitive function in patients with diabetes mellitus: guidance for daily care. Lancet Neurol..

[163] Gudala K., Bansal D., Schifano F., Bhansali A. (2013). Diabetes mellitus and risk of dementia: A meta-analysis of prospective observational studies. J. Diabetes Investig..

[164] Zhang J., Chen C., Hua S., Liao H., Wang M., Xiong Y., Cao F. (2017). An updated meta-analysis of cohort studies: Diabetes and risk of Alzheimer’s disease. Diabetes Res. Clin. Pract..

[165] Gaudieri P.A., Chen R., Greer T.F., Holmes C.S. (2008). Cognitive function in children with type 1 diabetes: A meta-analysis. Diabetes Care.

[166] Hughes T.M., Ryan C.M., Aizenstein H.J., Nunley K., Gianaros P.J., Miller R., Costacou T., Strotmeyer E.S., Orchard T.J., Rosano C. (2013). Frontal gray matter atrophy in middle aged adults with type 1 diabetes is independent of cardiovascular risk factors and diabetes complications. J. Diabetes Its Complicat..

[167] Ferguson S.C., Blane A., Wardlaw J., Frier B.M., Perros P., McCrimmon R.J., Deary I.J. (2005). Influence of an early-onset age of type 1 diabetes on cerebral structure and cognitive function. Diabetes Care.

[168] Biessels G.J., Despa F. (2018). Cognitive decline and dementia in diabetes mellitus: mechanisms and clinical implications. Nat. Rev. Endocrinol..

[169] Feinkohl I., Price J.F., Strachan M.W., Frier B.M. (2015). The impact of diabetes on cognitive decline: Potential vascular, metabolic, and psychosocial risk factors. Alzheimer’s Res. Ther..

[170] Geijselaers S.L.C., Sep S.J.S., Stehouwer C.D.A., Biessels G.J. (2015). Glucose regulation, cognition, and brain MRI in type 2 diabetes: a systematic review. Lancet Diabetes Endocrinol..

[171] Jeon B.T., Heo R.W., Jeong E.A., Yi C.O., Lee J.Y., Kim K.E., Kim H., Roh G.S. (2016). Effects of caloric restriction on O-GlcNAcylation, Ca(2+) signaling, and learning impairment in the hippocampus of ob/ob mice. Neurobiol. Aging.

[172] Liu Y.W., Zhu X., Yang Q.Q., Lu Q., Wang J.Y., Li H.P., Wei Y.Q., Yin J.L., Yin X.X. (2013). Suppression of methylglyoxal hyperactivity by mangiferin can prevent diabetes-associated cognitive decline in rats. Psychopharmacology.

[173] Bhutada P., Mundhada Y., Bansod K., Bhutada C., Tawari S., Dixit P., Mundhada D. (2010). Ameliorative effect of quercetin on memory dysfunction in streptozotocin-induced diabetic rats. Neurobiol. Learn. Mem..

[174] Maciel R.M., Carvalho F.B., Olabiyi A.A., Schmatz R., Gutierres J.M., Stefanello N., Zanini D., Rosa M.M., Andrade C.M., Rubin M.A. (2016). Neuroprotective effects of quercetin on memory and anxiogenic-like behavior in diabetic rats: Role of ectonucleotidases and acetylcholinesterase activities. Biomed. Pharmacother..

[175] Thakur A.K., Rai G., Chatterjee S.S., Kumar V. (2016). Beneficial effects of an Andrographis paniculata extract and andrographolide on cognitive functions in streptozotocin-induced diabetic rats. Pharm. Biol..

[176] Hashmi W.J., Ismail H., Mehmood F., Mirza B. (2018). Neuroprotective, antidiabetic and antioxidant effect of Hedera nepalensis and lupeol against STZ + AlCl3 induced rats model. Daru.

[177] Sanna R.S., Muthangi S., Devi S.A. (2019). Grape seed proanthocyanidin extract and insulin prevents cognitive decline in type 1 diabetic rat by impacting Bcl-2 and Bax in the prefrontal cortex. Metab. Brain Dis..

[178] Imam-Fulani A.O., Sanusi K.O., Owoyele B.V. (2018). Effects of acetone extract of Cola nitida on brain sodium-potassium adenosine triphosphatase activity and spatial memory in healthy and streptozotocin-induced diabetic female Wistar rats. J. Basic Clin. Physiol. Pharmacol..

[179] Thakur A.K., Chatterjee S.S., Kumar V. (2013). Beneficial effects of Brassica juncea on cognitive functions in rats. Pharm. Biol..

[180] Mousavi S.M., Niazmand S., Hosseini M., Hassanzadeh Z., Sadeghnia H.R., Vafaee F., Keshavarzi Z. (2015). Beneficial Effects of Teucrium polium and Metformin on Diabetes-Induced Memory Impairments and Brain Tissue Oxidative Damage in Rats. Int. J. Alzheimers Dis..

[181] Farajpour R., Sadigh-Eteghad S., Ahmadian N., Farzipour M., Mahmoudi J., Majdi A. (2017). Chronic Administration of Rosa canina Hydro-Alcoholic Extract Attenuates Depressive-Like Behavior and Recognition Memory Impairment in Diabetic Mice: A Possible Role of Oxidative Stress. Med Princ Pract..

[182] Lin W.S., Lo J.H., Yang J.H., Wang H.W., Fan S.Z., Yen J.H., Wang P.Y. (2017). Ludwigia octovalvis extract improves glycemic control and memory performance in diabetic mice. J. Ethnopharmacol..

[183] Liu Z.H., Chen H.G., Wu P.F., Yao Q., Cheng H.K., Yu W., Liu C. (2015). Flos Puerariae Extract Ameliorates Cognitive Impairment in Streptozotocin-Induced Diabetic Mice. Evid Based Complement Alternat Med..

[184] Parihar M.S., Chaudhary M., Shetty R., Hemnani T. (2004). Susceptibility of hippocampus and cerebral cortex to oxidative damage in streptozotocin treated mice: prevention by extracts of Withania somnifera and Aloe vera. J. Clin. Neurosci..

[185] Pandey S.P., Singh H.K., Prasad S. (2015). Alterations in Hippocampal Oxidative Stress, Expression of AMPA Receptor GluR2 Subunit and Associated Spatial Memory Loss by Bacopa monnieri Extract (CDRI-08) in Streptozotocin-Induced Diabetes Mellitus Type 2 Mice. PLoS ONE.

[186] Patel S.S., Udayabanu M. (2014). Urtica dioica extract attenuates depressive like behavior and associative memory dysfunction in dexamethasone induced diabetic mice. Metab. Brain Dis..

